# Impact of Male Infanticide on the Social Structure of Mountain Gorillas

**DOI:** 10.1371/journal.pone.0078256

**Published:** 2013-11-06

**Authors:** Andrew M. Robbins, Maryke Gray, Augustin Basabose, Prosper Uwingeli, Innocent Mburanumwe, Edwin Kagoda, Martha M. Robbins

**Affiliations:** 1 Department of Primatology, Max Planck Institute for Evolutionary Anthropology, Leipzig, Germany; 2 The International Gorilla Conservation Programme, Kigali, Kigali Province, Rwanda; 3 Parc National des Volcans, Rwanda Development Board, Kigali, Kigali Province, Rwanda; 4 Parc National des Virunga-sud, Institut Congolais pour la Conservation de la Nature, c/o International Gorilla Conservation Programme, Gisenyi, Western Province, Rwanda; 5 Bwindi Mgahinga Conservation Area, Uganda Wildlife Authority, Kampala, Kampala District, Uganda; Université de Strasbourg, France

## Abstract

Infanticide can be a major influence upon the social structure of species in which females maintain long-term associations with males. Previous studies have suggested that female mountain gorillas benefit from residing in multimale groups because infanticide occurs when one-male groups disintegrate after the dominant male dies. Here we measure the impact of infanticide on the reproductive success of female mountain gorillas, and we examine whether their dispersal patterns reflect a strategy to avoid infanticide. Using more than 40 years of data from up to 70% of the entire population, we found that only 1.7% of the infants that were born in the study had died from infanticide during group disintegrations. The rarity of such infanticide mainly reflects a low mortality rate of dominant males in one-male groups, and it does not dispel previous observations that infanticide occurs during group disintegrations. After including infanticide from causes other than group disintegrations, infanticide victims represented up to 5.5% of the offspring born during the study, and they accounted for up to 21% of infant mortality. The overall rates of infanticide were 2–3 times higher in one-male groups than multimale groups, but those differences were not statistically significant. Infant mortality, the length of interbirth intervals, and the age of first reproduction were not significantly different between one-male versus multimale groups, so we found no significant fitness benefits for females to prefer multimale groups. In addition, we found limited evidence that female dispersal patterns reflect a preference for multimale groups. If the strength of selection is modest for females to avoid group disintegrations, than any preference for multimale groups may be slow to evolve. Alternatively, variability in male strength might give some one-male groups a lower infanticide risk than some multimale groups, which could explain why both types of groups remain common.

## Introduction

Infanticide by males has been observed or suspected in a wide range of taxa including birds, carnivores, ungulates, and primates [1]–[6]. According to the sexual selection theory, infanticide can be an adaptive strategy for males when three conditions are fulfilled [7], [8]. The first condition is that the male had little or no probability of siring the infant. The second condition is that the mother will resume reproduction sooner if the infant is killed. The third condition is that the male has an increased probability of siring the mother’s next offspring [7], [8]. Several other hypotheses have been proposed to explain infanticide [9], [10].

Infanticide typically reduces the fitness of the mother, so females are expected to develop counterstrategies to avoid such losses [11]–[13]. After reviewing infanticide rates in twenty primate populations, Janson and van Schaik concluded that one of their main counterstrategies may be to encourage multimale grouping [14]. Polyandrous mating in multimale groups can enable a female to increase the number of potential fathers that will protect her offspring rather than trying to kill it, especially after the dominant male gets replaced [10], [15], [16]. The term “replacement” refers to a situation when the dominant male dies, or when he loses the dominant role to a subordinate within the group (internal takeover), or when he loses the dominant role to an outsider male (external takeover). An “outsider” male is a male that was outside the group when infants were sired within the group.

**Figure 1:**
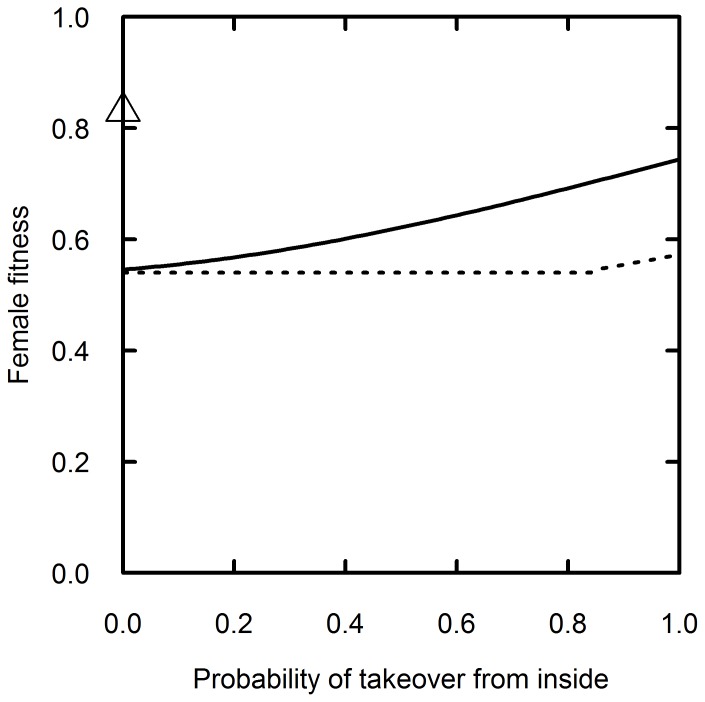
Predictions of the male strength model. Female fitness if the strength of dominant males is approximately three times higher in one-male groups (triangle) than in multimale groups (lines). Female fitness in multimale groups can depend on the probability that dominance takeovers will be done by insider males (rather than outsider males). Fitness can also depend on whether reproductive skew within multimale groups is primarily controlled by females (solid line) or the dominant male (dotted line). Inside takeovers and reproductive skew are impossible without subordinate males, so one-male groups are represented by a single point instead of two lines. Female fitness was consistently lower in multimale groups that had weaker dominant males than one-male groups. This graph is adapted from Figure 4 in reference [16].

In support of the comparative study by Janson and van Schaik [14], a mathematical model has predicted that females should generally prefer to live in multimale groups [16]. The predicted advantage of multimale groups diminished, however, when dominant males in both types of groups were much stronger than subordinates and solitary males. The study suggested that females could accept one-male groups under such conditions, and that females might even prefer one-male groups if multimale groups imposed any extra costs that were not incorporated into the model [16]. The model also indicated that females could prefer one-male groups if they have much stronger dominant males than multimale groups (Figure 1). Stronger males were given longer dominance tenures in the model, which offset the higher risk of infanticide when they were replaced in one-male groups [16]–[18]. We define the term “stronger” to represent males with longer dominance tenures (lower rates of replacement). We will refer to the mathematical model as the “male strength model” because it illustrates how variability in male strength could influence the impact of infanticide upon social structure.

This paper examines the potential impact of infanticide upon the social structure of mountain gorillas in the Virunga volcano region. We measure the impact of infanticide on the reproductive success of female mountain gorillas, and we examine whether their dispersal patterns reflect a strategy to avoid infanticide. Mountain gorillas are an interesting species for studies of infanticide because one-male and multimale groups are both common in the same population, and because infanticide has been common too [19], [20]. We define “multimale” to indicate a breeding group that has at least two males that are both at least 12 years old, the age at which they are considered adults or “silverbacks” [21]–[23]. Both males and females may be philopatric or disperse, so the reproductive strategies of each sex can directly influence social structure [23], [24]. Subordinate silverbacks emigrate to become solitary males, but those solitary males typically do not join breeding groups where the dominant male is alive (e.g., no external takeovers, [25], [26]). Females transfer directly to a solitary male or to another group [27]–[30].

The death of an infant mountain gorilla shortens the interval until next ovulation of the mother [31], [32], which fulfills one of the main criteria of the sexual selection hypothesis [8]. Genetic analyses indicate that subordinate males sired 15% of the gorillas born in multimale groups, and none of the 48 offspring that were tested were sired by males residing outside the group [33]. When subordinate males become dominant, they typically do not kill the infants within their group [33]. Infanticide by outsider males has been more common, particularly when a one-male group disintegrates following the death of the dominant male [20], [34]. The term “disintegrate” refers to a situation when all adult females (including females with unweaned offspring) join outsider males, thereby putting those offspring at a high risk of infanticide. Groups can also end through attrition (all members die or gradually leave the group), but due to the threat of infanticide, females with unweaned offspring rarely leave while the dominant male is alive [35], [36].

Despite the infanticide that occurs when one-male groups disintegrate, 50–60% of adult females reside in one-male groups, and mixed results have emerged from analyses of whether dispersing females prefer multimale groups [21], [28], [30], [37]. The results from mountain gorillas are not entirely comparable with published examples from the male strength model, which place more emphasis on dominance takeovers than dominant male mortality [16]. Nonetheless, the same general principles could apply: females could prefer one-male groups if they have stronger dominant males than multimale groups, because the longer dominance tenures in one-male groups could offset the higher risk of infanticide when those tenures ended [16]. Rates of dominant male mortality and replacements have not yet been reported for one-male versus multimale groups of mountain gorillas, however, so the male strength model has not been directly tested for this species.

An alternative explanation for the presence of females in one-male groups is the phylogenetic inertia hypothesis: the reproductive strategies of females may not yet fully reflect the benefits of multimale groups, which are considered a recent development in the evolutionary history of mountain gorillas [30], [38], [39]. Empirical results have supported the phylogenetic inertia hypothesis by showing significantly higher rates of infanticide and overall infant mortality in one-male groups than multimale groups [30], [40]. All of those results came from the southeastern sector of the Virungas, however, where multimale groups had an unusually large number of silverbacks in recent years [19], [41]. In addition, much of the data from one-male groups was collected during the early years of research when the population was declining due to relatively high levels of human disturbances [20], [21], [30]. Thus a more comprehensive study may be more representative of the evolutionary history of this species.

**Figure 2:**
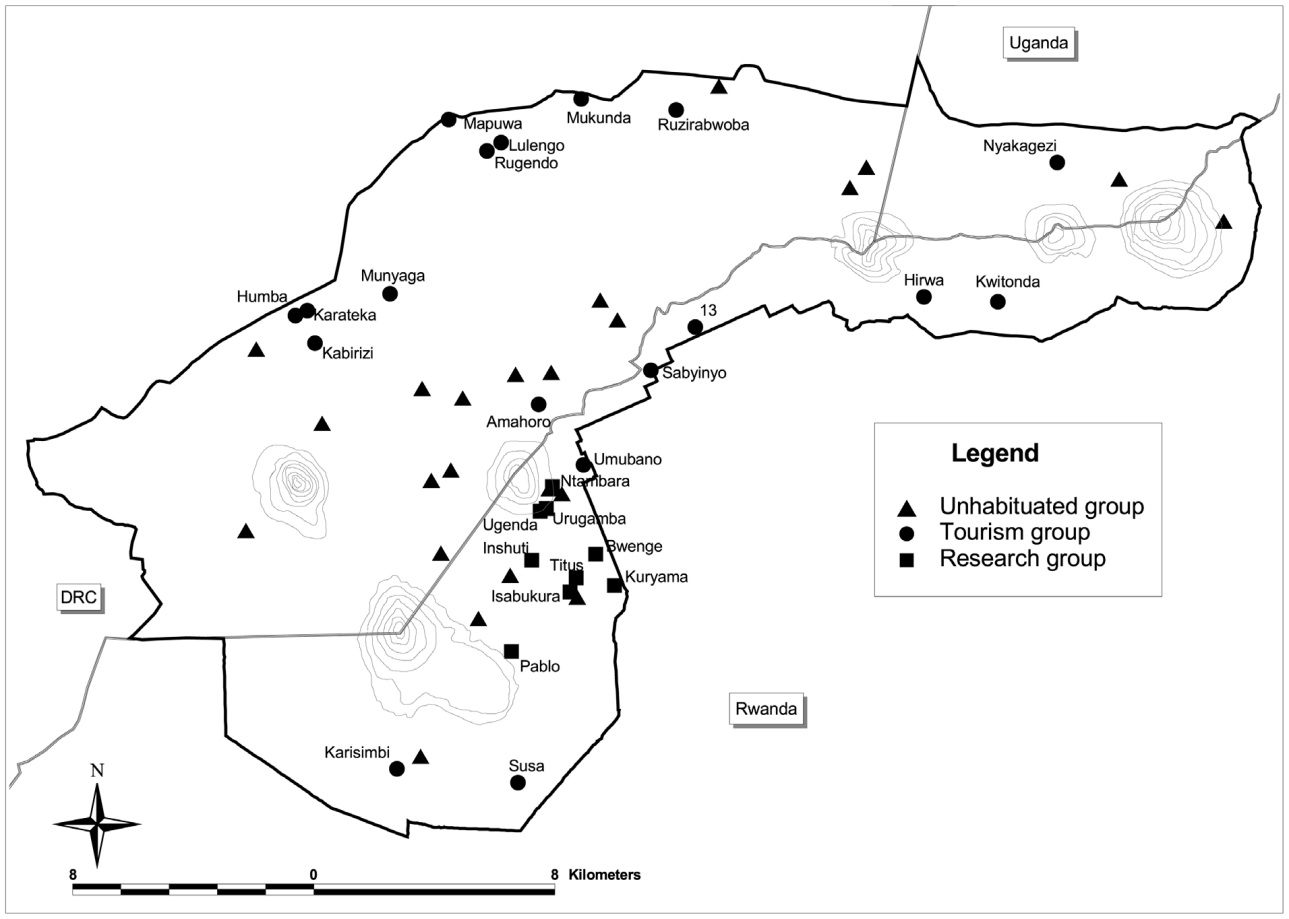
Spatial distribution of gorilla groups in the Virungas. Results of the 2010 census for groups in “new” ICGP dataset (tourist groups), the “previously published” Karisoke dataset (research groups), and groups that are not included in this study (unhabituated groups). The dark black line indicates park boundaries; the dark grey lines indicate international boundaries; and the light grey lines are contours of mountains.

This paper evaluates both the male strength model and the phylogenetic inertia hypothesis by combining previously published results with new data from one-male and multimale groups throughout the region (Figure 2). The combined dataset spans more than 40 years of observation and it comprises up to 70% of the entire population of Virunga mountain gorillas [19], [41], [42]. To test whether one-male groups have stronger dominant males than multimale groups, we compare the rates of dominant male mortality and replacements (mortality plus dominance takeovers) in each group type. To test whether infanticide is more frequent in one-male groups, we combine the previous results with new cases in each group type, including cases of infanticide that were caused by factors other than dominant male replacements. To test whether females *should* prefer multimale groups, we compare overall infant mortality and other measures of reproductive success in each group type. To test whether dispersing females actually *do* prefer multimale groups, we examine their probability of emigration and rates of immigration with each group type. We discuss how the results may help to explain the social structure of mountain gorillas, as well as the variability in infanticide rates among other species.

## Methods

### Ethics Statement

This research involved non-invasive work with wild non-human primates. All work was done in accordance with guidelines of the national authorities where the work occurred.

### Study Population and Data Collection

This study uses both new data and previously published results from studies of the Virunga mountain gorillas. The “new dataset” comes from the long term records of the International Gorilla Conservation Programme (IGCP), the Rwanda Development Board, the Institut Congolais pour la Conservation de la Nature, and the Uganda Wildlife Authority [42], [43]. The data are freely available from those agencies when reasonably requested for the purpose of academic, non-commercial research. Data were used for 21 social units (groups and solitary males) throughout the Virunga Volcano region from June 1979 through April 2010 (Table 1). Each of the habituated groups is continuously monitored by a separate team of observers, who are trained to systematically complete a standardized data sheet during every visit to the gorillas, with special emphasis on rapidly spotting when a gorilla is missing or injured [43]. Each habituated group is generally observed daily, but monitoring has occasionally been interrupted due to civil unrest, particularly during the mid-1990s [42].

**Table 1:** Summary of social units in the new dataset.

	First	Last	Total						Female	
Social unit	Year	Year	Years	gorillas	AF	SB	BB	%mmg	Years	Fate
Amahoro	1996	2010	14.1	15.7	5.7	2.0	1.6	69%	80.7	End of study
Buhanga	1998	2010	12.1	1.0	0.0	1.0	0.0	0%	0.3	End of study
Group11	1979	1993	13.6	10.2	2.4	2.2	1.6	53%	33.0	Disappeared
Group13	1979	2010	29.2	10.7	4.7	1.1	1.3	10%	135.8	End of study
Group9	1980	1992	12.4	8.2	2.9	1.0	2.0	0%	36.2	Disappeared
Hirwa	2006	2010	3.8	11.0	5.4	1.0	0.0	0%	20.7	End of study
Humba	1998	2010	11.8	10.3	3.7	1.4	1.6	44%	43.7	End of study
Kabirizi	1997	2010	12.5	28.3	10.4	1.1	2.2	7%	129.8	End of study
Karateka	1998	2010	12.2	1.0	0.0	1.0	0.0	0%	0.0	End of study
Karisimbi	2009	2010	0.8	14.6	3.6	3.0	2.6	100%	2.7	End of study
Kwitonda	1998	2010	12.2	14.2	4.3	1.5	1.9	27%	52.1	End of study
Lulengo	1998	2010	12.2	4.4	0.9	1.7	1.4	64%	11.3	End of study
Mapuwa	1995	2010	14.6	8.2	3.9	1.1	1.0	14%	56.8	End of study
Munyaga	1998	2010	12.5	6.4	0.9	2.3	2.4	51%	10.8	End of study
Nyakagezi	1998	2010	12.3	8.9	2.2	2.2	1.5	100%	27.3	End of study
PiliPili	2002	2009	6.5	1.3	0.2	1.0	0.0	0%	1.5	Lost all AF
Rugendo	1997	2010	12.5	9.2	3.2	1.8	1.6	53%	40.6	End of study
Ruzirabwoba	1995	2010	14.6	1.0	0.0	1.0	0.0	0%	0.0	End of study
Sabyinyo	1989	2010	20.9	9.4	3.3	1.9	1.4	73%	68.0	End of study
Susa Grp	1978	2010	31.7	28.0	9.5	2.9	2.8	96%	299.4	End of study
Umubano	2002	2010	7.9	7.7	2.7	1.0	1.0	0%	21.8	End of study
overall	1978	2010	280.1	11.2	3.8	1.6	1.5	39%	1072.1	

First, last, and total years of observation for each group and/or solitary male. The composition includes the average number of gorillas, adult females (AF), silverbacks (SB), and blackbacks (BB); as well as the percentage of observation months in which the social unit was multimale (%mmg). Female-years equal the combined number of days that each female an adult during the study, divided by 365.25.

The “combined dataset” combines the new data with previously published results from 1967–2007 that are primarily from the Karisoke Research Center [44]. The previous results have been reported in sufficient detail for the analyses of dominant male mortality and replacement rates [25], [26], [33], [45], female fertility and offspring survival [40], natal female transfers versus philopatry [29], secondary transfers by parous females [30], and immigration by all females [30]. Previously published studies often include at least one group from the long term records of the IGCP [20], [28], [30], [31], [40], so we have made appropriate adjustments to avoid double-counting such data. Sample sizes may vary among our analyses because the previously published results often span different time intervals.

Gorillas are considered infants until they reach age three (the typical age of weaning), and then juveniles until they reach age six, and then subadults until they reach age eight. Immatures are defined as the sum of infants, juveniles, and subadults [19]. Females are considered adults when they reach age eight. After reaching age eight, males are called blackbacks until they reach age twelve, and those 12+ years old are called adult males or silverbacks [22]. The precision of birthdates is estimated to be within a few months for gorillas that were first observed as infants, and within 1–2 years for the gorillas that were first observed as they approached adulthood, and within 4–10 years for gorillas that were first observed as adults [22].

### Dominant Male Mortality Rates and Replacement Rates

We used rate-based χ^2^ calculations to compare the mortality rates for dominant males in one-male versus multimale groups [46]. The mortality rate equaled the number of dominant males that died in each group type, divided by the number of group-years that were observed in each group type [14]. The expected number of deaths was proportional to the number of group-years that each type was observed. Group-years equal the number of elapsed days from the beginning to the end of observations, divided by 365.25. We also used rate-based χ^2^ calculations to compare the rates of dominant male replacements (deaths plus takeovers) in each group type. The replacement rate equaled the number of dominant male replacements in each group type, divided by the number of group-years that were observed in each group type. For both rate-based χ^2^ calculations, we report a separate set of p-values for analyses that excluded five poaching deaths. All analyses excluded males that did not remain dominant long enough to sire any offspring (e.g., solitary males that acquired females for a few days or months before losing them again).

### Rates of Infanticide and their Components

Following Janson and van Schaik [14], we define the infanticide rate as the total number of infanticides (*I_T_*) divided by the number of births (*B*). The total number of infanticides can be separated into the number of infanticides due to dominant male replacements (*I_R_*) plus the number of infanticides due to other causes (*I_O_*). Several studies have also reported the (relative) infanticide rate as the total number of infanticides (*I_T_*) divided by the total cases of infant mortality (*M_T_*), so we present results from that perspective too [14], [20], [47]. See Table 2 for a summary of the abbreviations used for parameters in this text.

**Table 2:** Comparisons between one-male groups versus multimale groups.

Parameter	Description	OMG	MMG	Overall
*yr*	group-years observed	152	178	330
*D*	dominant male deaths	5	11	16
*R*	dominant male replacements	5	16	21
*B*	total births	145	199	344
*P_R_*	infants present during replacements	7	50	57
*I_R_*	infanticide cases due to replacements	4	3	7
*I_D_*	infanticide cases due to disintegrations	4	2	6
*I_O_*	infanticide not due to replacements	8	4	12
*I_T_*	total cases of infanticide	12	7	19
*I_S_*	strong cases of infanticide	7	3	10
*M_T_*	total infant mortality	41	48	89
*M_O_*	mortality not due to infanticide	29	41	70
*D/yr*	dominant male mortality rate	0.033	0.062	0.048
*R/yr*	dominant male replacement rate	0.033	0.090	0.064
*P_R/_B*	proportion of infants present during replacements	4.8%	25.1%	16.6%
*I_R/_P_R_*	probability of infanticide during a replacement	57.1%	6.0%	12.3%
*I_R/_B*	rate of infanticide due to replacements	2.8%	1.5%	2.0%
*I_D_/B*	rate of infanticide due to disintegrations	2.8%	1.0%	1.7%
*I_D_/M_T_*	disintegration cases versus overall infant mortality	9.8%	4.2%	6.7%
*I_O_/B*	rate of other infanticides	5.5%	2.0%	3.5%
*I_T_/B*	overall infanticide rate	8.3%	3.5%	5.5%
*I_T_/M_T_*	total infanticide versus overall infant mortality	29.3%	14.6%	21.3%
*I_S_/B*	infanticide rate for strong cases only	4.8%	1.5%	2.9%
*I_S_/M_T_*	strong cases versus overall infant mortality	17.1%	6.3%	11.2%
*M_T_/B*	total mortality rate	28.3%	24.1%	25.9%
*M_O/_(B-I_T_)*	mortality rate excluding infanticide	21.8%	21.4%	21.5%

Data are from this study and one other study [40]. The “Overall” column represents combined results from one-male groups (OMG) plus multimale groups (MMG). The term “replacement” refers to a situation when the dominant male dies, or when he loses the dominant role to a subordinate within the group (internal takeover), or when he loses the dominant role to an outsider male (external takeover). See Methods for the criteria for “strong” versus “total” cases of infanticide.

One of the main hypotheses of this study is that dominant males may have longer tenures in one-male groups, which could offset the higher risk of infanticide when those tenures end (Figure 1). The components of this hypothesis are represented by Equation 1:
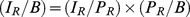
(1)where (*I_R_/B*) represents the rate of infanticide due to replacements. *P_R_* equals the number of infants who were present during replacements, so (*I_R_*/*P_R_*) equals the proportion of those infants who were killed during replacements. *P_R_*/*B* reflects the probability that an offspring will be present as an infant during a dominant male replacement. *P_R_*/*B* which will typically be low when dominance tenures are long, because most offspring will have enough time to complete infancy before the dominance tenure ends (see Section S4 in File S1 for a more detailed analysis). Thus, long dominance tenures (leading to low values of *P_R_*/*B)* could give one-male groups a low infanticide rate (*I_R_/B*) despite a high risk of infanticide when those tenures end (*I_R_*/*P_R_*). Figure S1 and Section S8 in File S1 provide a graphical description of Equation 1 and a hypothetical example of its relevance to our hypothesis.

We used Fisher exact tests to compare *I_R_*/*P_R_*, *P_R_*/*B, M_T_/B,* and the rates of infanticide in one-male versus multimale groups. Our analyses of infanticide rates and their components exclude offspring born during the last three years of observations because their survival through infancy could not be fully evaluated (e.g., as in [40]). Evidence for infanticide was considered “strong” if an observer witnessed an attack that resulted in the wounding or death of an infant, or if observers found an infant’s body with bite wounds and/or other wounds after a known encounter between social units or merger of two groups [20]. The disappearance of an apparently healthy infant was considered “possible” evidence of infanticide if it coincided with a known encounter with an outsider male [20]. See Section S1 and Table S1 in File S1 for details about infanticide cases that have not been previously reported.

### Female Reproductive Success (Other than the Impact of Offspring Mortality)

To examine whether females should prefer multimale groups for reasons other than offspring mortality, we first performed a t-test to compare the age of first reproduction (AFR) for females whose first offspring was born in one-male versus multimale groups. Our analyses of the AFR were limited to data points in which the age of the mother and her first offspring were both in known to within 15 days.

As an additional assessment of whether females should prefer multimale groups for reasons other than offspring mortality, we ran generalized linear mixed models (GLMM) to compare the length of their interbirth intervals (IBI) in each group type. Those analyses were limited to data points in which the offspring survived to reach age three, and the beginning and end of the interval were both known to within 15 days. The identity of the mother was included as a random effect variable to control for multiple sampling of the same adult females. We also included a fixed effect variable to control for parity, because primiparous mothers have shown lower reproductive success than multiparous females [32]. Statistical analyses of AFR and IBI were limited to the new dataset because we did not have enough details to incorporate previously published data.

### Female Dispersal

To assess whether dispersing females prefer multimale groups, we first examined the probability of dispersal for natal nulliparous females in each group type. The analysis was limited to nulliparous females that made a natal transfer or gave birth in their natal group during the study [29]. We defined the transfer percentage as the number of transfers, divided by the number of transfers plus births. We used a Fisher exact test to compare the frequency of transfers versus births in each group type [29].

As a second assessment of whether dispersing females prefer multimale groups, we examined the probability of dispersal for parous females in each group type. The analysis was based on the number of transfers and the number of births by parous females during the study [30]. We again defined the transfer percentage as the number of transfers, divided by the number of transfers plus births. We used a Fisher exact test to compare the frequency of transfers versus births in each group type [30]. Due to the risk of infanticide, females rarely transfer while pregnant or lactating, so most transfers occur while the female is cycling [35], [36]. Thus, by comparing the relative frequency of emigrations versus births, the analyses are essentially comparing the number of emigrations versus the number of time intervals when females may have opportunities for transferring (time periods when females are cycling).

As a third assessment of whether dispersing females prefer multimale groups, we examined the rates of immigration by all females (nulliparous and parous) into each group type. For each group type, the immigration rate equals the number of females that immigrated, divided by the number of group-years that the group type was observed [30]. The rate-based χ^2^ calculations compared the actual number of female immigrations into each group type versus the expected number of those immigrations [46]. The expected number of immigrations was based on the null hypothesis that they would be distributed in proportion to the number of group-years that each group type was observed (i.e., the two group types would have the same immigration rate, [30]). We assume that females will be more likely to immigrate into the type of group that they prefer. The immigration rate of each group may also depend on other factors such as the number of females in neighboring groups, but such information was not available for this study. Similarly, our analyses of immigration rates did not distinguish between natal nulliparous females versus parous females because those classifications were not known for all immigrants [30].

### Statistical Methods

We performed the rate-based χ^2^ calculations in an Excel spreadsheet, the GLMM and Fisher exact tests with R (http://www.R-project.org), and the t-tests using Systat 11 (2004, SYSTAT Software Inc., Richmond CA). See Section S6 in File S1 for additional analyses that compare the rate-based χ^2^ calculations and Fisher exact tests with GLMM, as well as analyses that compare the reliability of the new dataset versus previously published results.

## Results

### Dominant Male Mortality Rates and Replacement Rates

The male strength model and prior studies of this population have both highlighted the risk of infanticide when dominance tenures end, so we examined the mortality rates and replacement rates (mortality plus takeovers) of dominant males in one-male versus multimale groups [16], [20]. During the 330 group-years that dominant males were observed in one-male and multimale groups (combined), their mortality rate was 0.048 deaths per group-year and their replacement rate was 0.064 replacements per group-year (Table 2). The mortality rate of dominant males was 0.033 deaths per group-year in one-male groups, which is not significantly different from 0.062 deaths per group-year in multimale groups (rate-based χ^2^ = 1.4, df = 1, p = 0.23; without poaching: p = 0.063). The replacement rate of dominant males was 0.033 replacements per group-year in one-male groups, which is significantly lower than 0.090 replacements per group-year in multimale groups (rate-based χ^2^ = 4.3, df = 1, p = 0.04; without poaching: p = 0.007). See Section S2 and Table S3 in File S1 for additional details about the fate of each group after the death of the dominant male.

### Infanticide due to Dominant Male Replacements

Offspring that were born in a one-male group had a 4.8% probability of being present as infants during a dominant male replacement (*P_R_/B*), which is significantly lower than the 25.1% probability for offspring born in multimale groups (Fisher exact test: p<0.001). When infants were present during a dominant male replacement, they had a 57.1% probability of being killed in one-male groups (*I_R_/P_R_*), which is significantly higher than the 6.0% probability of infanticide in multimale groups (Fisher exact test: p = 0.0026). After using Equation 1 to combine those two comparisons, we found that infants born in one-male groups had a 2.8% probability of being killed due to dominant male replacements (*I_R_/B*), which is not significantly different from the 1.5% probability for infants born in multimale groups (Fisher exact test: p = 0.46). Thus for infants in one-male groups, the lower probability of being present during a dominant male replacement (*P_R_/B*) partially offset the higher risk of infanticide when those replacements occurred (*I_R_/P_R_*). See Sections S3 & S4 in File S1 for additional details about the rates of infanticide due to dominant male replacements.

Of the seven cases of infanticide that occurred during dominant male replacements, one occurred during a group fission and the other six occurred during group disintegrations. The six cases from disintegrations represent 1.7% of infants born in the study groups, and 6.7% of infant mortality (Table 2). One of the disintegrations involved a “borderline” multimale group that was close to being a one-male group (i.e., the oldest subordinate was near the transition from a blackback into a silverback). In addition, four infants were not killed when their groups disintegrated, but those cases also involved marginal circumstances that do not dispel previous observations that infanticide will typically occur during group disintegrations (e.g., the infant was nearly weaned [20]). See Sections S3 & S5 in File S1 for more details about these marginal situations.

### Infanticide due to causes other than Dominant Male Replacements

In addition to the seven cases of infanticide that were due to replacements of the dominant male, there were twelve other strong or possible cases of infanticide among offspring born in the study groups when infant survival could be evaluated (Table 2). Eight of those twelve cases (67%) resulted from encounters between two social units while their dominant silverbacks were alive. One of the other infants was killed by gorilla(s) within its own group, and the context is unknown for the other three cases (but they did not coincide with a replacement). Collectively, those twelve cases represent 5.5% of the offspring born in one-male groups, which is not significantly different from the 2.0% for offspring born in multimale groups (Fisher exact test: p = 0.13). See Section S1 in File S1 for details about the new infanticide cases that were not due to dominant male replacements.

### Overall Rates of Infanticide and Infant Mortality

The nineteen strong and possible cases of infanticide represent 5.5% of the offspring born during the study, and they accounted for 21.3% of infant mortality (Table 2). Those nineteen cases accounted for 29.3% of infant mortality in one-male groups, which is not significantly different from 14.6% in multimale groups (Fisher exact test: p = 0.12). The ten strong cases of infanticide accounted for 17.1% of infant mortality in one-male groups, which is not significantly different from 6.3% in multimale groups (Fisher exact test: p = 0.18). See Table S2 in File S1 for more details about the nineteen strong and possible cases of infanticide.

Of the 344 offspring born during this study, 89 died in infancy, so overall infant mortality equaled 25.9%. Infant mortality was 28.3% for offspring born in one-male groups, which is not significantly different from 24.1% for offspring born in multimale groups (Fisher exact test: p = 0.39). Hypothetically, the comparison of overall infant mortality would not be significant if the higher rates of infanticide in one-male groups were offset by higher infant mortality from other causes in multimale groups. After excluding both strong and possible cases of infanticide, however, infant mortality was almost identical in one-male versus multimale groups (21.8% versus 21.4%, Fisher exact test: p>0.99). Thus we found no evidence that overall infanticide rates or infant mortality differed significantly between one-male versus multimale groups.

### Female Reproductive Success (Other than the Impact of Offspring Mortality)

To examine whether females should prefer multimale groups for reasons other than offspring mortality, we first compared their age of first reproduction (AFR) in each group type. Among the twenty females in the new dataset with precisely known birthdates for themselves and their first offspring, the AFR averaged 9.9±2.2 years. The AFR was 9.6±3.2 years for six females in one-male groups, which is not significantly different from 10.0±1.8 years for fourteen females in multimale groups (t = 0.38, df = 18, p = 0.71).

As an additional assessment of whether females should prefer multimale groups for reasons other than offspring mortality, we compared the length of their interbirth intervals (IBI) in each group type. Among the groups in the new dataset, the average length of interbirth intervals was 3.9±0.75 years for 51 IBI in one-male groups, versus 4.1±1.1 years for 53 IBI in multimale groups. The average length was 4.5±1.0 years for 21 IBI by primiparous females, versus 3.9±0.92 years for 83 IBI by parous females. After controlling for parity and the identity of the mother, the difference between one-male versus multimale groups was not statistically significant (GLMM: t = 1.9, standard error = 0.20, p_MCMC_ = 0.37). Thus these results for AFR and IBI provide no significant evidence that female mountain gorillas should prefer multimale groups for reasons other than offspring mortality.

### Female Dispersal

To assess whether females actually do prefer multimale groups, we first examined the probability of dispersal for natal nulliparous females from each group type. Of the 75 natal nulliparous females in the combined dataset, 40% stayed and reproduced in their natal group. The other 60% transferred before reproducing, so the transfer percentage among all natal nulliparous females was 60%. The transfer percentage was 74% for the 23 natal nulliparous females in one-male groups, which is not significantly different from 54% for the 52 natal nulliparous females in multimale groups (Fisher exact test: p = 0.13).

As a second assessment of whether females prefer multimale groups, we examined the probability of dispersal for parous females from each group type. In the combined dataset, parous females gave birth 341 times and they transferred 101 times. Thus their total number of births and transfers was 442 (341+101), and their transfer percentage was 23% (101/442). In one-male groups, parous females gave birth 144 times and made 68 voluntary transfers, which represents a transfer percentage of 32%. In multimale groups, parous females gave birth 197 times and made 33 voluntary transfers, represents a transfer percentage of 14%. The transfer percentage of parous females was significantly higher in one-male groups than in multimale groups (Fisher exact test: p<0.001). Thus, parous females were significantly more likely to leave one-male groups than multimale groups.

As a third assessment of whether dispersing females prefer multimale groups, we compared their rates of immigration into each group type. During the 162.8 group-years that one-male groups were observed, 63 females immigrated, which represents a rate of 0.39 immigrations per group-year. During the 187.4 group years that multimale groups were observed, 60 females immigrated, which represents a rate of 0.32 immigrations per group-year. The difference between those two immigration rates is not statistically significant (rate-based χ2 = 1.1, df = 1, p = 0.292). Collectively, the analyses of dispersal provided mixed results regarding whether female mountain gorillas prefer multimale groups.

## Discussion

Strong and possible cases of infanticide accounted for 21% of infant mortality in this study, so infanticide may be a major contributor to infant mortality among the Virunga mountain gorillas [20], [40]. Polyandrous mating in multimale groups is reportedly one of the main counterstrategies for females to avoid infanticide [10], [15], so why do 50–60% of adult female mountain gorillas reside in one-male groups? We discuss two hypotheses and we compare our results with other primate populations.

### Differences in Dominant Male Strength

Based on the “male strength model”, our first hypothesis was that females may prefer one-male groups that have “stronger” dominant males than multimale groups [16]. Stronger dominant males were defined to have lower rates of “replacement”, which refers to instances when the dominant male dies, and when he loses the dominant role to a subordinate male or an outsider male (a.k.a. an internal or external takeover). In this study, the replacement rate for dominant males was almost three times higher in multimale groups than one-male groups, which resembles the differences in strength that were needed for females to prefer one-male groups in the male strength model (Figure 1). Pradhan and van Schaik proposed that differences in male strength could arise in species with high predation [16], but mountain gorillas currently have no natural predators. Variations in male strength could also depend on other environmental factors, genetics, and age [48]–[50]. See Section S7 in File S1 for potential explanations about how such variations could lead to stronger dominant males in one-male groups than in multimale groups.

Although one-male groups had lower rates of dominant male replacements, their rates of infanticide were still 2–3 times higher than in multimale groups during this study. Those differences in infanticide rates were not statistically significant, and they were in the opposite direction of examples from the male strength model, which showed that lower replacement rates could lead to lower rates of infanticide in one-male groups than multimale groups (Figure 1). The contrast between our results and the model may partially arise from the low rate of infanticide when infants were present during dominant male replacements in multimale groups in this study (*I_R_/P_R_* = 6% in Table 2). Subordinate silverbacks sire only 15% of the offspring born in multimale groups [33], but there are several potential reasons why they typically do not commit infanticide when they become dominant. Firstly, the inclusive fitness benefits of infanticide would be reduced if the subordinate male was related to the dominant silverback [51]. Secondly, subordinates may have a higher than 15% probability of siring infants during a gradual replacement process (such as an internal takeover that occurs over the course of months or years), although this explanation would not apply when the dominant male was killed by poachers. Thirdly, the optimal male strategies for infanticide may not have evolved, especially because females can manipulate the information that males use to estimate paternity probabilities by concealing ovulation and mating with more than one male [17], [52], [53].

Even if the overall risks of infanticide are higher in one-male groups than multimale groups, we cannot exclude the possibility that differences in silverback strength might give some one-male groups a lower infanticide risk than some multimale groups. If so, then variability in silverback strength could help to explain why mountain gorillas have both types of groups [20], [27]. Larger silverbacks had lower offspring mortality in groups of western lowland gorillas [54], which may reflect differences in strength, but similar studies are needed to compare one-male versus multimale groups of mountain gorillas. See Section S7 in File S1 for additional discussion about the social structure of western gorillas versus mountain gorillas.

### Phylogenetic Inertia

An alternative explanation for the presence of females in one-male groups is the phylogenetic inertia hypothesis: the reproductive strategies of females (e.g., dispersal) may not yet fully reflect the benefits of multimale groups [30], [38], [39]. Parous females were significantly more likely to leave one-male groups than multimale groups in this study. Nonetheless, the probability of emigration from one-male groups was only 32% for parous females, so the other 68% of the parous females in one-male groups stayed and reproduced despite higher rates of infanticide. Furthermore, the difference between one-male versus multimale groups was not significant for the emigration of natal nulliparous females or for the immigration rates of all females. Differences in emigration frequencies could be influenced by greater herding by resident males in multimale groups that prevent females from emigrating [29], [30], [55]. The dispersal of natal nulliparous females may also be influenced by inbreeding avoidance, although some females might gain inclusive fitness benefits by inbreeding if the relatedness is less than 0.5 (i.e., not breeding with fathers or full brothers, see [29], [56], [57]). Thus the dispersal data continues to provide limited evidence that female mountain gorillas prefer multimale groups.

Overall infant mortality was not significantly higher in one-male groups (28%) than multimale groups (24%) during this study. In contrast, infant mortality was significantly higher in one-male groups (42%) than multimale groups (22%) during a previous study of a subset of this population [40]. Thus, in comparison with the previous study, our more comprehensive results suggest weaker selection pressures for females to prefer multimale groups, which may explain why the evidence for such preferences is also weak. Results for the age of first reproduction and interbirth intervals during this study also showed no significant fitness benefits for females to reside in multimale groups.

Multimale groups are considered a recent development in the evolutionary history of mountain gorillas because their close taxonomic relatives (lowland gorillas) have very few multimale groups, because female gorillas do not have conspicuous sexual swellings or long estrous periods, and because silverbacks do not have large testes [58], [59]. If those physical adaptations to multimale groups have not yet evolved in this species, then the evolution of female preferences for multimale groups may also be incomplete. In contrast, phylogenetic inertia was not considered an important factor in a comparative study of infanticide rates, because the greatest variations were found within species rather than among species [14].

### Comparisons with Other Studies

Strong cases of infanticide represented 11% of infant mortality and 2.9% of the 344 births in this study. The updated proportions of infanticide are lower than an initial assessment of this population, when strong cases represented 37% of infant mortality and 14% of the 50 births [20]. Most strikingly, strong cases that followed the death of a dominant male declined from 12% of births in the earlier analyses to just 1.7% in this study. The updated results reflect a lower rate of group disintegrations, mainly due to a lower mortality rate for dominant males in one-male groups, so they do not dispel previous observations that infanticide will typically occur during such disintegrations (See Section S3 in File S1). High variability in infanticide rates have also been observed in other primates, which illustrates the importance of long-term studies even in species where research is well-established [10], [60], [61].

The reduced rate of infanticide in the Virungas is consistent with preliminary results from Bwindi mountain gorillas, where the dominant male replacement rate has been even lower (0.055 replacements per group-year at Bwindi, versus 0.064 replacements per group-year in this study), and no infanticide has been reported among the 51 infants observed [62]. Almost 50% of groups have been multimale at Bwindi [63]. Among western gorillas at Mbeli, nine infants disappeared following the death of dominant males, which represents 12% of all observed births [64], and is significantly higher than 1.7% in the same context during this study (Fisher exact test, p<0.001). Although field methods at Mbeli are not well suited for detecting infanticide in other contexts, the greater impact of dominant male deaths at Mbeli (versus our study) is consistent with the absence of multimale groups among western gorillas [64]. In contrast with Mbeli, infanticide has not been reported following the death of eight dominant males among eastern lowland gorillas at Kahuzi-Biega, which also have almost no multimale groups [65]. Infants survived in groups without a silverback for up to 29 months, despite possible encounters with outsider males up to several times per month [66]. Only three cases of infanticide have been reported in Kahuzi-Biega, and all were associated with female transfers between surviving silverbacks [67]. The contrast between close taxonomic relatives such as eastern versus western lowland gorillas may highlight the potential challenges of understanding differences among species that are not closely related.

Although infanticide was a major contributor to overall infant mortality in this study, its frequency was lower than some other primate populations, where it has accounted for 30–70% of infant mortality [47]. Dominant male mountain gorillas also had the lowest replacement rates in a comparison of 20 primate populations, but those rates have not shown a straightforward relationship with the frequency of infanticide, even after controlling for the length of interbirth intervals when infants would be vulnerable [14]. Another potentially important factor may be the proportion of dominant male replacements that involve outsider males [16]. In groups where data for infant survival was available, outsider male mountain gorillas were associated with only 5 of the 21 replacements of dominant silverbacks in this study (24%), and the dominant silverbacks had died in all five of those cases.

Previous studies of mountain gorillas have suggested that outsider males do not overtake a dominant silverback because the females could simply leave the intruder [20], [59]. Nonetheless, outsider takeovers and high rates of infanticide occur in other species with female dispersal, including dispersal to avoid incursions by outsider males [47], [68], [69]. Two other potential explanations are life histories and population density. The slower life history of mountain gorillas may increase the potential importance of long reproductive lifespans, so outsider males may prefer to acquire females gradually (via transfers) as a less risky strategy than completely defeating a dominant silverback [20]. And while some primate groups encounter outsider males almost daily, such encounters only occur monthly for mountain gorillas, so it may be easier for dominant silverbacks to avoid conflicts [55], [70]–[73]. High population density has been linked to higher rates of male takeovers and infanticide in blue monkeys and langurs [74], [75]. If the rate of infanticide varies with population density, then its influence on population dynamics may rival the importance of food availability and predation in some species [14], [42], [76].

## Supporting Information

File S1 **Supporting Information.** Additional details about the new and previously reported cases of infanticide, about the proportion of infants that were present during replacements, and about the fate of infants when the dominant male dies. Sensitivity analyses for the two datasets and the statistical methods used in this study.
(DOC)
