# Supplementary material for: Impact of Male Infanticide on the Social Structure of Mountain Gorillas
Source: PLoS One. 2013 Nov 6;8(11):e78256. doi: 10.1371/journal.pone.0078256 (PMC3819382; doi:10.1371/journal.pone.0078256)

Supporting Information

Impact of male infanticide

on the social structure of mountain gorillas

Section S1. New cases of infanticide

Section S2. Dominant male tenures and group fates

Section S3. Fate of infants during dominant male replacements

Section S4. Relative proportion of infants who were present during a replacement

Section S5. Sensitivity analyses with "borderline" one-male/multimale groups

Section S6. Sensitivity analyses of the statistical methods and datasets

Section S7. Additional discussion points

Section S8. Additional descriptions of Equation 1 from the main text

References

Table S1. New cases of infanticide

Table S2. Infanticide cases when overall infant mortality could be evaluated

Table S3. Fate of each group following the death of the dominant male

Figure S1. The rate of infanticide due to dominant male replacements

*Section S1. New cases of infanticide*

This section describes ten cases of infanticide that have not been previously reported (Table S1). Three of the ten new cases are based on physical evidence. The victim in case 1 was killed by a blow to her abdomen that ruptured her stomach, one kidney and bruised her intestines. Cases 2 & 3 were described as "trauma" and "severe internal trauma to head and lower body" respectively. In case 4, the dominant male died and the multimale group fissioned while the mother was pregnant. The offspring was killed within days after birth, even though the new dominant male had been in the same group as the mother when the infant was sired. The new dominant male did not kill an infant who was born two months earlier, and no infanticide was reported when a Karisoke group fissioned after the death of its dominant male [1, 2]. Case 4 was the only one of the ten new cases that coincided with the replacement of a dominant male.

Cases 5-10 are suspected infanticides because the infant disappeared during an encounter with an outsider male. Eight of the ten new cases (strong and suspected) coincided with encounters between social units, including four cases in which the female transferred (Table S1). The mother transferred with her infant in Cases 6 & 8; the infant disappeared during the encounter in Case 7; and it is unknown whether the transfer occurred before, during, or after the infanticide in Case 2. In Cases 2, 6, & 7 the female transferred again before reproducing (returning to her previous group in Cases 2 & 6), so it is unlikely that she reproduced with the potentially infanticidal male. In Case 8, we do not know which male committed the infanticide, and the group subsequently fissioned before the mother reproduced again [3]. Overall, these cases provide little evidence that the potentially infanticidal males reproduced with the mother of the victim. In case 4, however, the female stayed in the new group after the fission, so infanticide would have enabled the new dominant male to reproduce sooner.

When the ten new cases in Table S1 are combined with previously published results [2, 4, 5], there are now 15 strong cases and 15 suspected cases of infanticide reported among the Virunga mountain gorillas (two unsuccessful attempts are excluded). Nineteen of those cases occurred in study groups when data for overall infant mortality is available, and were counted in analyses of infanticide rates throughout this study (Table S2).

*Section S2. Dominant male tenures and group fates*

This section provides additional details about the tenures of dominant males and about the fate of each group (and its infants) after the death of the dominant male. Of the 45 dominant silverbacks in the combined dataset, 17 were already dominant when habituated, and 24 were still dominant when the studies ended. Of the 28 dominance tenures that were observed to begin, 32% started when a subordinate inherited a group following the death of the previous dominant male, 29% started when a group fissioned, 21% started when an all-male group or solitary male acquired females, and 18% started when a subordinate took over the dominant role. Of the 21 dominance tenures that were observed to end, 76% ended when the dominant male died, and the other 24% ended when he was overtaken by a subordinate within the group.

As stated in the main text, the replacement rate of dominant males was 0.064 replacements per group-year. The inverse of the replacement rate equals the mean tenure length for the full dataset, but that relationship does not apply separately to one-male and multimale groups because some silverbacks were dominant in both group types [6, 7]. We also used Kaplan Meier analyses with the full dataset to estimate the mean tenure length of males who were observed since they became dominant (i.e., not left censored). The mean tenure length was 15.7 years based on the inverse of the replacement rate, and 12.0 years based on Kaplan-Meier analysis.

In 7 of the 16 cases when the dominant male died (44%), the oldest subordinate was at least 14 years old, so the group had clearly been multimale (Table S3). The group remained stable in five of those cases, and it fissioned into two stable groups in each of the other two cases. In two cases when the dominant male died (12%), the oldest subordinate was less than 10 years old, so the group had clearly been one-male. Both of those groups disintegrated. Thus, when groups were clearly one-male or multimale, their fates were consistent with previous reports that disintegrations will occur after the dominant silverback dies in one-male groups but not multimale groups [4, 5].

In the remaining seven "borderline" cases (44%), the oldest subordinate was within ±two years of the transition between blackbacks and silverbacks (age 12) when the dominant male died, so the group was near the boundary between one-male versus multimale groups (Table S3). In one of those cases, all four adult females and their infants were killed by poachers at the same time as the dominant male, and the remaining males eventually joined an outsider male. One group remained stable. Two groups gradually lost all of their adult females without any cases of infanticide. Those two cases are not considered disintegrations because the females did not leave until their offspring were no longer infants. The other three groups disintegrated. Infanticide occurred during two of the three disintegrations, and a 34 month old infant survived the other one. Among these seven "borderline" cases, the oldest subordinate had an estimated age as high as 12.4 years old in the groups that disintegrated, and a known age as low as 10.8 years old in the groups that did not. Thus, the fates of groups and their infants were less predictable when the oldest subordinate was near the transition between being a blackback and a silverback after the dominant male died. See Section S5 in this Supporting Information for sensitivity analyses of the "borderline" cases.

*Section S3. Fate of infants during dominant male replacements*

This section provides additional details about the fates of infants who were present during dominant male replacements. As noted in the main text, infants had a 57.1% probability of being killed if they were present during a dominant male replacement in one-male groups (*I_R_/P_R_*). Three infants were not killed even though they were in one-male groups when the dominant male died. One of those infants was nearly weaned at 36 months of age [5], one died from pneumonia before its mother encountered another group [5], and the third infant was in a group that remained stable. When the dominant male died in the group that remained stable, the oldest subordinate male was within one year of becoming a silverback, so the group was close to being considered a multimale group. Thus all three of these cases could be considered to have extenuating (or marginal) circumstances.

As stated in the main text, 6% of the infants who were present during a dominant male replacement in a multimale group were killed (Table S3). One infant was killed by a male (Bm) who was present when the infant was born, but the male had uncharacteristically immigrated as a blackback [4]. Another infant was soon killed by an outsider male when Bm could not maintain the group and it disintegrated [4]. Both of the first two cases were in a "borderline" multimale group, because Bm has been described as either a blackback or a silverback at the time [4, 5, 8]. The third (possible) case among multimale groups occurred when a group fissioned after the death of the dominant male (Case #4 in Table S1).

No cases of infanticide have been reported among the ~16 infants who were present during an internal takeover of the dominant silverback by a resident subordinate. In addition, two infants did not die from infanticide when a borderline multimale group disintegrated after the death of its dominant silverback ("Munane" in Table S3). One of those infants (Gasindikira) was 34 months old when an outsider male took over the group, and the other infant (Gwira) had already died from pneumonia.

Of the ten infants who were present during a group disintegration, six died from infanticide, two died from other causes before their mother joined an outsider male, and the other two were near the boundary between infants and juveniles (36 months old). Thus, all four cases without infanticide could be considered to have extenuating (or marginal) circumstances, so they do not dispel previous observations that infanticide will typically occur during group disintegrations. Instead, our main finding is that group disintegrations are rare, primarily due to the relatively low mortality rate of dominant males in one-male groups (see main text).

*Section S4. Relative proportion of infants who were present during a replacement*

This section expands upon Equation 1 in the Methods of the main text, to provide a more detailed analysis of how dominant male replacements may influence infanticide rates in one-male versus multimale groups. As explained in the main text, an infant's probability of being present during a dominant male replacement was more than five times higher in multimale groups (*P_R_/B* = 25.1%) than in one-male groups (*P_R_/B* = 4.8%). The replacement rate of dominant males was less than three times higher in multimale groups (0.090 replacements per group-year) than in one-male groups (0.033 replacements per group-year). Thus the three-fold difference in replacement rates (between multimale versus one‑male groups) may not fully explain the five-fold difference in the probability that offspring were infants during a dominant male replacement (*P_R_/B*). To further compare the replacement rates with (*P_R_/B*), we specify that:

*Z = (P_R_/B) / (R/yr)* Equation S1

Z can be considered the *relative* proportion of offspring who were infants during a replacement, because it equals the *actual* proportion (*P_R_/B*), *relative to* the replacement rate (*R/yr*). Smaller values of Z may indicate that relatively few of a dominant male's offspring were born shortly before he was replaced (e.g., if females anticipated the replacement and left to reproduce elsewhere). Larger values of Z may indicate that a relatively high proportion of a dominant male's offspring were born shortly before he was replaced (e.g., if a male gradually accumulated more females until he was suddenly replaced). *Z*-values could also be larger in species with a longer period of infancy, so any comparisons among species may require additional adjustments (similar to the "relative replacement rates" in [9]).

In this study, the value for *Z* was almost twice as high in multimale groups (2.8) as in one‑male groups (1.5). Although further study is needed to understand those results, it would not be surprising if females are more careful to avoid reproducing with dominant males who are "weakening" in one-male groups than in multimale groups, because the risk of infanticide is so much greater when the male gets replaced in a one‑male group. Regardless, the empirical effect of those observed differences in Z‑values during this study was to make infanticide less likely in one-male groups (compared with multimale groups) than would be expected based on replacement rates.

After combining Equation S1 with Equation 1 from the Methods of the main text, we get an expanded expression for the rates of infanticide due to replacements:

(*I_R_/B*) = (*R/yr)* x *Z* x (*I_R_*/*P_R_*) Equation S2

As with Equation 1, (*I_R_/B*) represents the proportion of offspring who died from infanticide due to dominant male replacements. Those infanticide rates depend on the replacement rates of dominant males (*R/yr)*, the relative probability that infants will be present during those dominant male replacements (*Z*), and the risk that infants will die from infanticide if they are present during a dominant male replacement (*I_R_/P_R_*). Equation S2 incorporates the replacement rates more explicitly than Equation 1, so it may provide a framework for expanding a comparative study of infanticide rates and replacement rates among many primate populations [9]. In addition to comparing one‑male versus multimale groups within a population (as done in this study), researchers could also compare overall values for each parameter throughout each population, which would help to pinpoint the proximate causes for differences in the infanticide rates among species.

*Section S5. Sensitivity analyses with "borderline" one-male/multimale groups*

The distinction between one-male and multimale groups has traditionally been based on whether the oldest subordinate has reached age 12 (see the Methods section in the main text). In this study, 35% of the infanticide and 44% of the dominant male deaths occurred when the oldest subordinate was within ±two years of that age. In such "borderline" cases, the dichotomous distinction between one-male versus multimale groups may not fully reflect a more continuous variation in the maturation of those subordinates from blackbacks into silverbacks. To examine whether our main conclusions were sensitive to those variations, we ran multiple permutations of those analyses while assuming that each borderline case could be either a one-male group or a multimale group. For example, there were seven borderline cases for the fate of a group when the dominant male died (See Section S2 of this Supporting Information), so we ran 2^7^ = 128 permutations to examine every possible combination of one-male and multimale groups from those cases.

As stated in the main text, the replacement rate of dominant males was 0.033 replacements per group-year in one-male groups, which is significantly lower than 0.090 replacements per group-year in multimale groups (rate based chi‑square = 4.3, df = 1, p = 0.04). When we assumed that each of the seven "borderline" subordinates could be either a blackback or a silverback, the replacement rate remained lower for one-male groups than multimale groups in all 128 permutations, but the difference was not statistically significant in half of those permutations. Thus we had mixed results regarding whether one-male groups have significantly lower replacement rates than multimale groups.

As stated in the main text, the nineteen strong and possible cases of infanticide accounted for 29.3% of infant mortality in one-male groups, which is not significantly different from 14.6% in multimale groups (Fisher exact test: p = 0.12). The ten strong cases of infanticide accounted for 17.1% of infant mortality in one-male groups, which is not significantly different from 6.3% in multimale groups (Fisher exact test: p = 0.18).

When we assumed that each borderline case could be in either a one-male or a multimale group, the infanticide rates were significantly different in 18% of permutations for strong cases and 11% of permutations for strong and possible cases. Thus a large majority of those sensitivity analyses indicate that rates of infanticide were not significantly higher in one‑male groups than multimale groups.

As stated in the main text, infant mortality was 28.3% for the 145 offspring born in one‑male groups, which is not significantly different from 24.1% for the 199 offspring born in multimale groups (Fisher exact test: p = 0.39). Even if we assume that all "borderline" groups were one-male whenever disintegrations occurred, infant mortality still would not be significantly higher in one-male groups than multimale groups (p = 0.26). Thus, even if one-male groups have significantly higher infanticide rates than multimale groups (previous paragraph), the impact of infanticide was not large enough to create significant differences in overall infant mortality.

Collectively, these sensitivity analyses illustrate that the statistical significance of analyses are more tentative when p-values near 0.05, especially when sample sizes are small. The permutation tests showed mixed results when the p-value was near 0.05 in the base case (the analyses of replacement rates), but the results remained consistent when the p-value was far from 0.05 in the base case (the analyses of infant mortality). Sample sizes were also smaller for the analyses of replacement rates (N = 21 dominant male replacements) than for the analyses of infant mortality (N=89 deaths among 344 infants). The sample sizes, p-values, and permutation results for infanticide rates fell in between those other two sets of analyses.

*Section S6. Additional sensitivity analyses of the statistical methods and datasets*

As explained in the Methods, we used a Fisher exact test to compare the overall infant mortality in one-male versus multimale groups. To test whether those results depend upon the identity of each female, we replaced the Fisher exact test with a generalized linear mixed model (GLMM) using the identity of the adult female as a random effect variable. The p-values for infant mortality remained similar (not shown), which suggests that the identity of the adult female is not an important variable in our analyses. Our comparison was limited to the new dataset because the random effect variable was not available from previously published data [2, 10]. GLMM with the previously published data have also shown that the identity of the adult female is not an important variable in our analyses of female emigration (unpublished results).

Similarly, we replaced the rate-based chi-squared test for dominant male mortality with a GLMM using random effect variables for the group ID, the sectors of the study area, and the ID of each dominant male. Group type (one-male versus multimale) was the fixed effect variable. The analysis included one data point for each year that each male was dominant. The response variable equaled "1" if the dominant male died and "0" if he survived. In addition to running the full model with all three random effect variables, we also ran reduced models that excluded any one or two of those variables. The p‑values for group type were essentially identical in all of those GLMM (not shown), which suggests that the random effect variables had minimal influence on our analyses.

To compare the reliability of the new dataset versus previously published results, we analyzed two parameters that would be most sensitive to missed observations: interbirth intervals (IBI) and immigration rates. It is not visually apparent when mountain gorillas are pregnant, which increases the possibility that observers can miss infants that disappear immediately after birth [11]. Such missed infants would make IBI seem deceptively long (because the previous IBI would seem to continue), so a dataset with longer IBI might have more missed infants. The length of IBI averaged 4.0 ± 0.75 years in a previous study [2], which is essentially identical to 4.0 ± 0.97 years in the new dataset (N = 88 & 104 IBI respectively, t=0.13, p=0.89). Hypothetically, observers could also miss females that leave shortly after immigrating, so a study with a lower immigration rate may indicate that more female transfers were missed. The immigration rate was 0.35 during 147.0 group-years in a previous study [10], which is essentially identical to 0.35 during 203.2 group-years in the new dataset (χ2 = 0.008, df = 1, p = 0.93). Thus we conclude that the two datasets are equally reliable.

*Section S7. Additional discussion points*

This section contains two elaborates on two issues that arose in the Discussion of the main text: why would one-male groups have stronger dominant males than multimale groups, and why are multimale groups so rare among western gorillas?

As described in the main text, the replacement rate for dominant males was significantly higher in multimale groups than one male groups, which indicates that one‑male groups have "stronger" dominant males according to our definition of the term. One-male groups could have stronger dominant males than multimale groups for multiple reasons that are not mutually exclusive. If females avoid social units where the only silverback is weak, then those silverbacks will be solitary males or in non‑breeding groups rather than in one-male breeding groups (See Section S4 in the Supporting Information, as well as [5, 12-14]). If subordinates males emigrate from multimale groups where the dominant male is strong, then those groups may become one‑male groups [5, 15-17]. Mountain gorillas have an age-graded social structure, so if multimale groups have older dominant males than one-male groups, and if males weaken as they get older, then multimale groups will have weaker dominant males than one-male groups [6, 8, 18-21]. Further study is needed to quantify the proximate mechanisms for one-male groups to have stronger dominant males than multimale groups.

As we mentioned in the Discussion, multimale groups are common among mountain gorillas but not western gorillas. One potential explanation would be if western gorilla groups were not large enough to retain more than one adult male [16]. Multimale groups of primates are typically larger than one‑male groups because subordinate males are more likely to stay in groups that contain more females [18, 22, 23]. Average group sizes do not vary significantly among gorilla populations, however, so any ecological constraints on the group size of western gorillas may not fully explain why they lack multimale groups [24].

A second potential explanation is that the one-male group structure of western gorillas may be perpetuated by an interaction between male and female dispersal strategies [16]. If subordinate male western gorillas always emigrate to become solitary, then females cannot develop a preference for multimale groups, because such groups never exist. If female western gorillas have no preference for multimale groups, then philopatry may not be beneficial for males. The explanation is similar to the phylogenetic inertia hypothesis for mountain gorillas. It has been further proposed that these self-perpetuating interactions were interrupted among mountain gorillas when human disturbances induced some males to become philopatric [16].

A third potential explanation has been that the risk of infanticide among western gorillas may be low even without multimale groups, if neighboring groups form dispersed networks of related males whose incentive to kill infants is reduced by inclusive fitness [16, 25]. Anecdotal reports indicate that some infants have survived disintegrations of western gorilla groups despite joining an outsider male [26, 27]. Nonetheless, the rate of infanticide due to group disintegrations was significantly higher among western gorillas at Mbeli than in this study, so the dominant males and adult females of western gorillas could potentially have higher fitness if they had multimale groups (see Discussion). Thus the ultimate reasons for differences in social structure between western gorillas and mountain gorillas have not been fully explained.

*Section S8. Additional descriptions of Equation 1*

This section provides some more basic explanations of Equation 1 from the main text. The equation describes the rate of infanticide that occurs due to dominant male replacements. The rate equals the number of infanticides due to replacements (*I_R_*) divided by the number of births (*B*). Dividing by *B* can help to compare data obtained from different levels of sampling. For example, if a 20‑year study observed 1000 births and 40 infanticides, then its infanticide rate would be 0.04 infanticides per birth. If a one‑year study observed 50 births and 30 infanticides, then its infanticide rate would be 0.60 infanticides per birth. The rates suggest that infanticide may be more important in the population with a one-year study, even though it had a smaller number of total cases than the 20-year study.

Figure S1 illustrates some examples from Equation 1. The y-axis represents the rate of infanticide due to dominant male replacements (*I_R_/B*), and the x-axis represents the proportion of infants during dominant male replacements that were killed (*I_R_*/*P_R_*). Each line represents a constant value for (*P_R_*/*B*), which equals the proportion of offspring that were present as infants during dominant male replacements. The lines have an upward slope, which shows that at each value of (*P_R_*/*B*), the infanticide rate increases when infants who are present during a replacement have a higher probability of getting killed (*I_R_*/*P_R_*). At each value of the x-axis, the differences among lines show that the infanticide rate increases when more infants are present during replacements (*P_R_*/*B*).

One of the main hypotheses of this study is that dominant males may have longer tenures in one-male groups, which could offset the higher risk of infanticide when those tenures end (see Figure 1 in the main text). The enlarged symbols in Figure S1 illustrate such a (hypothetical) scenario, which demonstrates the biological relevance of the terms in Equation 1. The enlarged square represents one-male groups, and the enlarged triangle represents multimale groups. Dominance tenures are longer in the one-male groups than in multimale groups, as represented by the lower proportion of infants that are present during a replacement (*P_R_*/*B)* in one‑male groups (5%) than in multimale groups (25%). The risk of infanticide for offspring who are present during a replacement (*I_R_/P_R_*) is higher in one-male groups (90%) than in multimale groups (30%). The rate of infanticide due to replacements (*I_R_/B*) is lower in one‑male groups (4.5%) than in multimale groups (7.5%). Thus the dominance tenures in one‑male groups are long enough to offset the higher risk of infanticide when those tenures end.

**References**

1. Robbins MM (2001) Variation in the social system of mountain gorillas: the male perspective. In: Robbins MM, Sicotte P, Stewart KJ, editors. Mountain gorillas: three decades of research at Karisoke. Cambridge: Cambridge University Press. pp. 29-58.

2. Robbins MM, Robbins AM, Gerald-Steklis N, Steklis HD (2007) Socioecological influences on the reproductive success of female mountain gorillas (*Gorilla beringei beringei*)*.* Behavioral Ecology and Sociobiology 61(6): 919-931.

3. Stoinski TS, Vecellio V, Ngaboyamahina T, Ndagijimana F, Rosenbaum S, et al. (2009) Proximate factors influencing dispersal decisions in male mountain gorillas, *Gorilla beringei beringei.* Animal Behaviour 77(5): 1155-1164.

4. Fossey D (1984) Infanticide in mountain gorillas (*Gorilla gorilla beringei*) with comparative notes on chimpanzees. In: Hausfater G, Hrdy S, editors. Infanticide: comparative and evolutionary perspectives. New York: Aldine, Hawthorne. pp. 217-236.

5. Watts DP (1989) Infanticide in mountain gorillas - new cases and a reconsideration of the evidence*.* Ethology 81(1): 1-18.

6. Dunbar R (1984) Reproductive decisions: an economic analysis of Gelada baboon social strategies. Princeton: Princeton University Press. 265 p.

7. Makarieva AM, Gorshkov VG (2004) On the dependence of speciation rates on species abundance and characteristic population size*.* Journal of Biosciences 29(1): 119-128.

8. Robbins MM (1995) A demographic analysis of male life history and social structure of mountain gorillas*.* Behaviour 132: 21-47.

9. Janson CH, van Schaik CP (2000) The behavioral ecology of infanticide by males. In: Janson CH, van Schaik CP, editors. Infanticide by males and its implications. Cambridge: Cambridge University Press. pp. 469-494.

10. Robbins AM, Stoinski TS, Fawcett KA, Robbins MM (2009) Socioecological influences on the dispersal of female mountain gorillas - evidence of a second folivore paradox*.* Behavioral Ecology and Sociobiology 63(4): 477-489.

11. Robbins AM, Robbins MM, Gerald-Steklis N, Steklis HD (2006) Age-related patterns of reproductive success among female mountain gorillas*.* American Journal of Physical Anthropology 131(4): 511-521.

12. Caillaud D, Levrero F, Gatti S, Menard N, Raymond M (2008) Influence of male morphology on male mating status and behavior during interunit encounters in western lowland gorillas*.* American Journal of Physical Anthropology 135(4): 379-388.

13. Breuer T, Robbins AM, Boesch C, Robbins MM (2012) Phenotypic correlates of male reproductive success in western gorillas*.* Journal of Human Evolution 62(4): 466-472.

14. Sterck EHM (1997) Determinants of female dispersal in Thomas langurs*.* American Journal of Primatology 42(3): 179-198.

15. Robbins AM, Robbins MM (2005) Fitness consequences of dispersal decisions for male mountain gorillas (*Gorilla beringei beringei*)*.* Behavioral Ecology and Sociobiology 58(3): 295-309.

16. Harcourt AH, Stewart KJ (2007) Gorilla society: conflict, compromise, and cooperation between the sexes. Chicago: University of Chicago Press. 459 p.

17. Watts DP (2000) Causes and consequences of variation in male mountain gorilla life histories and group membership. In: Kappeler PM, editor. Primate males. Cambridge: Cambridge University Press. pp. 169-180.

18. Alberts SC, Watts HE, Altmann J (2003) Queuing and queue-jumping: long-term patterns of reproductive skew in male savannah baboons, *Papio cynocephalus.* Animal Behaviour 65: 821-840.

19. Robbins MM, Robbins AM (2004) Simulation of the population dynamics and social structure of the Virunga mountain gorillas*.* American Journal of Primatology 63(4): 201-223.

20. Parnell RJ (2002) Group size and structure in western lowland gorillas (*Gorilla gorilla gorilla*) at Mbeli Bai, Republic of Congo*.* American Journal of Primatology 56(4): 193-206.

21. Eisenberg JF, Muckenhirn NA, Rudran R (1972) Relation between Ecology and Social Structure in Primates*.* Science 176(4037): 863-864.

22. Altmann SA (1962) A field study of the sociobiology of rhesus monkeys, *Macaca mulatta*. Annual Proceedings of the New York Academy of Sciences 102: 338-435.

23. Lindenfors P, Froberg L, Nunn CL (2004) Females drive primate social evolution*.* Proceedings of the Royal Society of London Series B-Biological Sciences 271: S101-S103.

24. Yamagiwa J, Kahekwa J, Basabose AK (2003) Intra-specific variation in social organization of gorillas: implications for their social evolution*.* Primates 44(4): 359-369.

25. Bradley BJ, Doran-Sheehy DM, Lukas D, Boesch C, Vigilant L (2004) Dispersed male networks in western gorillas*.* Current Biology 14(6): 510-513.

26. Stokes EJ, Parnell RJ, Olejniczak C (2003) Female dispersal and reproductive success in wild western lowland gorillas (*Gorilla gorilla gorilla*)*.* Behavioral Ecology and Sociobiology 54(4): 329-339.

27. Genton C, Cristescu R, Gatti S, Levrero F, Bigot E, et al. (2012) Recovery Potential of a Western Lowland Gorilla Population following a Major Ebola Outbreak: Results from a Ten Year Study*.* Plos One 7(5).

Table S1. New cases of infanticide for offspring born in one-male groups (omg) and multimale groups (mmg). Contextual details include whether the supporting evidence is strong, whether dominant male had died (DomDied?), whether the mother transferred to another male (MomXfer?), and whether the infanticidal male may have reproduced with the mother (Icide success).

|  |  |  | Group |  | Death | Death | Dom | Mom | Icide |
| --- | --- | --- | --- | --- | --- | --- | --- | --- | --- |
| Case | Infant | Group | Type | Evidence | Date | Age | Died? | Xfer? | Success |
| 1 | Umurage | Amahoro | mmg | Strong | 26-Nov-08 | 2.44 | no | no | no |
| 2 | Ingabo | Susa Grp | omg | Strong | 13-Mar-91 | 0.20 | no | yes | no |
| 3 | Amizero | Group13 | omg | Strong | 6-May-89 | 0.01 | no | no | no |
| 4 | UmwumbINF | Umubano | omg | Possible | 14-Dec-02 | 0.00 | yes | no | yes |
| 5 | Impuruza | Sabyinyo | mmg | Possible | 17-Mar-03 | 2.73 | no | no | no |
| 6 | Assan | Humba | omg | Possible | 8-Mar-04 | 1.00 | no | yes | no |
| 7 | Sebiheri | Mapuwa | omg | Possible | 3-Feb-07 | 0.42 | no | yes | no |
| 8 | Infant72 | Susa Grp | mmg | Possible | 27-Jun-07 | 0.50 | no | yes | yes |
| 9 | IntambwINF | Group13 | omg | Possible | 23-Feb-08 | 0.25 | no | no | no |
| 10 | MagayanINF | Hirwa | omg | Possible | 12-Jan-09 | 0.27 | no | no | no |

Table S2. Summary of new and previously reported cases of infanticide in the study groups while overall infant mortality could be evaluated. Group types include one-male groups (omg) and multimale groups (mmg). The context includes group disintegrations (disint), and intergroup encounters (IGE). References are a=[4], b=[5], c=[2], d=This study.

|  |  |  | Group |  |  | Death |  |
| --- | --- | --- | --- | --- | --- | --- | --- |
| Case | Infant | Group | Type | Context | Evidence | Date | ref |
| F1 | Thor | Group8 | omg | disint | Strong | 19-May-74 | a |
| F2 | Frito | Group4 | mmg | disint | Strong | 14-Aug-78 | a |
| F3 | Mwelu | Group4 | mmg | disint | Strong | 5-Dec-78 | a |
| I | Kazi | Nunkie | omg | disint | Strong | 1-Jun-85 | b |
| II | Nirere | Nunkie | omg | disint | Strong | 1-Jul-85 | b |
| III | Karudi | Nunkie | omg | disint | strong | 1-Jul-85 | b |
| 4 | UmwumbINF | Umubano | omg | fission | possible | 14-Dec-02 | d |
| F4 | Curry | Group5 | omg | IGE | strong | 14-Apr-74 | a |
| 2 | Ingabo | Susa Grp | omg | IGE | strong | 13-Mar-91 | d |
| 1 | Umurage | Amahoro | mmg | IGE | strong | 26-Nov-08 | d |
| X | Petinf | Nunkie | omg | IGE | possible | 1-Jun-85 | b |
| 5 | Impuruza | Sabyinyo | mmg | IGE | possible | 17-Mar-03 | d |
| 6 | Assan | Humba | omg | IGE | possible | 8-Mar-04 | d |
| 7 | Sebiheri | Mapuwa | omg | IGE | possible | 3-Feb-07 | d |
| 8 | Infant72 | Susa Grp | mmg | IGE | possible | 27-Jun-07 | d |
| F5 | Banjo | Group5 | omg | internal | possible | 1-Mar-76 | a |
| 3 | Amizero | Group13 | omg | unknown | strong | 6-May-89 | d |
| a | krc01 | unknown | mmg | unknown | possible | unknown | c |
| b | krc02 | unknown | mmg | unknown | possible | unknown | c |

Table S3. Fate of each group following the death of the dominant male ("Domale"), including five cases in which he was killed by poachers ("Poach"). The data is sorted by the age of the oldest subordinate ("MaxSub"), and the "Precise" column shows the estimated precision of those ages (in years). The group type is classified as a multimale group (mmg) when it contains a subordinate over 12 years old, and as a one-male group (omg) when it doesn't. Those classifications are considered borderline when the subordinate is within ±two years of age 12, the transition between blackbacks and silverbacks. Group fates are classified as disintegrations ("disint"), declines, or stable; and one group (Rugendo) lost all of its adult females to poachers. We show the number of infants in the group when the dominant male died ("infant"), and the number of infants who were killed by infanticide ("icide"). The "dset" column indicates whether the data is previously published ("k") or new to this study ("t"). See the Results and the Supporting Information for additional details.

| Group | Year | Domale | Poach | MaxSub | Age | Precise | Border | Type | Fate | infant | icide | dset |
| --- | --- | --- | --- | --- | --- | --- | --- | --- | --- | --- | --- | --- |
| Tiger | 1987 | TG | no | -- | --- | --- | no | omg | disint | 1 | 0 | k |
| Nunkie | 1985 | NK | no | BB | 7.0 | <0.1 | no | omg | disint | 4 | 3 | k |
| Group13 | 1992 | Mrithi | yes | Munane | 10.8 | <0.1 | yes | omg | decline | 0 | 0 | t |
| Group13 | 1979 | Sbuname | yes | Mrithi | 11.9 | <0.1 | yes | omg | stable | 1 | 0 | t |
| Group8 | 1974 | RF | no | PN | 12.0 | 2 | yes | omg | disint | 1 | 1 | k |
| Group4 | 1978 | UB | yes | BM | 12.0 | 2 | yes | mmg | disint | 2 | 2 | k |
| Group13 | 2002 | Munane | no | Nyak | 12.4 | <0.1 | yes | mmg | disint | 2 | 0 | t |
| Group11 | 1980 | Stilgar | no | Ndume | 12.5 | <0.1 | yes | mmg | decline | 1 | 0 | t |
| Rugendo | 2007 | Senk | yes | Mukunda | 12.6 | <0.1 | yes | mmg | poached | 0 | 0 | t |
| Group4 | 1967 | WH | no | UB | 15.0 | 2 | no | mmg | stable | 2 | 0 | k |
| Munyaga | 2008 | Munyaga | no | Mawazo | 16.3 | <0.1 | no | mmg | stable | 0 | 0 | t |
| Amahoro | 2002 | Amh | no | Ubumwe | 17.4 | <0.1 | no | mmg | fission | 4 | 1 | t |
| Group5 | 1993 | ZZ | no | PB | 18.0 | <0.1 | no | mmg | fission | 7 | 0 | k |
| Rugendo | 2001 | Rugendo | yes | Senk | 18.6 | <0.1 | no | mmg | stable | 2 | 0 | t |
| Group5 | 1982 | IC | no | BV | 30.0 | 10 | no | mmg | stable | 2 | 0 | k |
| Susa | 1990 | IMB | no | JP | 30.9 | 2 | no | mmg | stable | 12 | 0 | t |

Figure S1: The rate of infanticide due to dominant male replacements (*I_R_/B*) versus the proportion of offspring that were present as infants during dominant male replacements (*P_R_*/*B*), and the probability that those infants would be killed if they were present during a replacement (*I_R_*/*P_R_*). Each line represents a constant value for (*P_R_*/*B*). The enlarged symbols highlight two data points that are discussed in the Section S8 of the Supporting Information.


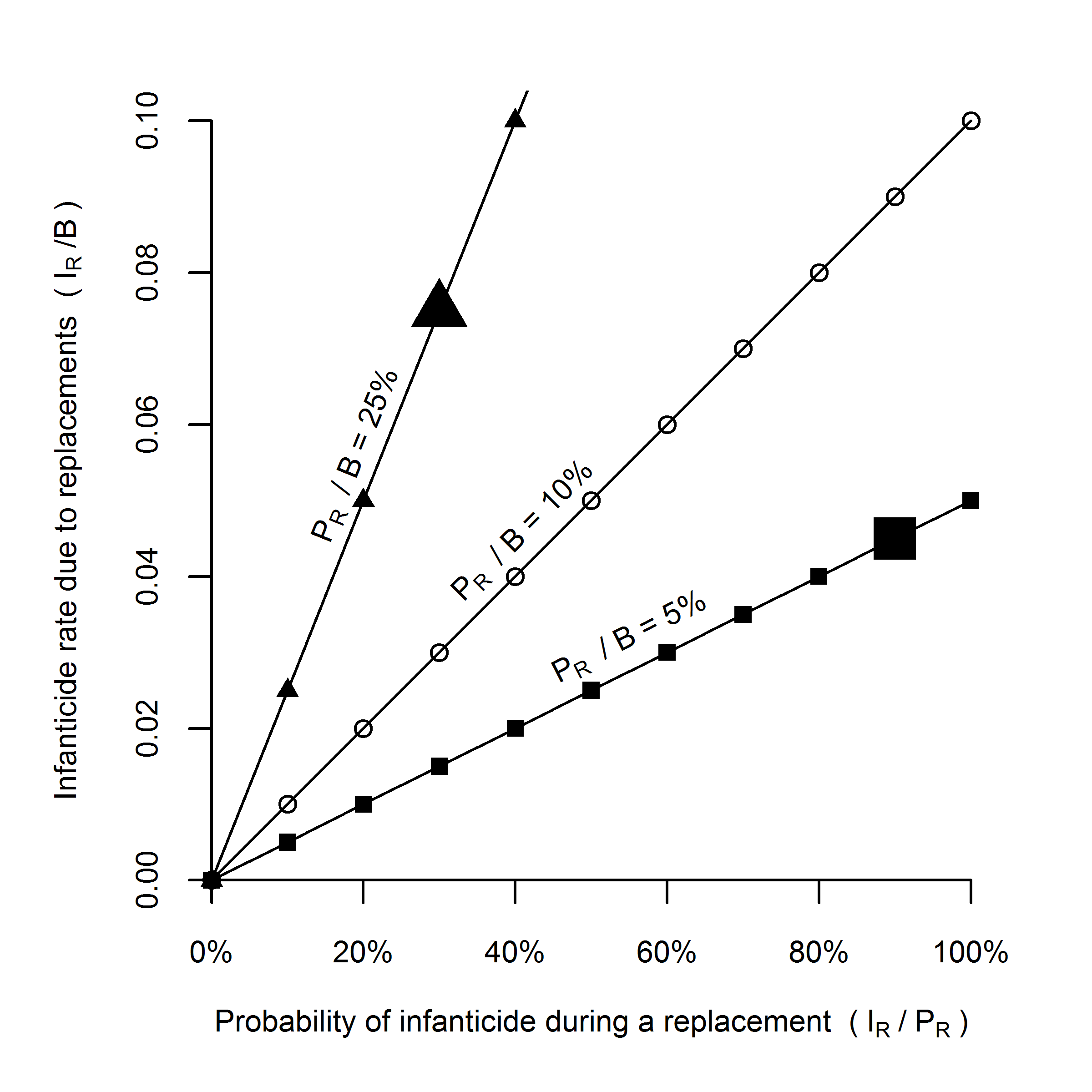

Supplement: File S1 — Supporting Information. Additional details about the new and previously reported cases of infanticide, about the proportion of infants that were present during replacements, and about the fate of infants when the dominant male dies. Sensitivity analyses for the two datasets and the statistical methods used in this study. (DOC) [file pone.0078256.s001.doc]
